# Notes and News

**Published:** 1896-02-29

**Authors:** 


### Notes and News.

(Collected and Communicated.)

HOSPITAL MEETINGS.

A Festival Dinner on behalf of the Royal Hospital for Diseases of the Chest will be held at Whitehall Rooms, the Hotel Metropole, on May 26th, under the presidency of Lord Charles Bruce.

An Anniversary Banquet of the Homes for Little Boys, Farningham and Swanley, will be held at Whitehall Rooms on March 19th, under the presidency of Sir George Newnes.

A Quarterly and Special General Court of Governors of the London Hospital will be held at the hospital, on Wednesday, March 4th, at two p.m.

Dr. Granville Bantock, F.R.C.S.Ed., senior surgeon to the Samaritan Hospital, London, having resigned, has been elected on the consulting staff.

Mr. George Richmond has contributed £1,000 to St. Thomas's Hospital, to endow a surgical bed "In memoriam" of James Richmond.

The annual general meeting of Governors of the National Hospital for Diseases of the Heart and Paralysis will be held at the hospital, on Wednesday, March 4th, at three p.m.

The Queen has personally decorated Surgeon-Captain Hilliard with the order of St. Michael and St. George. Surgeon-Captain Hilliard attended Prince Henry of Battenberg on his homeward journey until his death.

The new Dental Hospital for Ireland, situated in Dublin, has just been opened by the Lord Mayor of that city. The whole building is not yet complete, £6,000 being still required for the purpose. On the occasion of the opening the Duchess of Abercorn laid the foundation-stone of the new wing. The hospital, which owes its existence to the energy of Dr. Stack and to leading dental practitioners in Dublin, contains laboratory, museum, operation, anæsthetic, and recovery rooms. The new wing will contain lecture theatre and other departments.

A festival dinner in aid of the Victoria Hospital for Children will be held at the Whitehall Rooms, Hotel Metropole, on Saturday, February 29th, when H.R.H. the Duke of York will be in the chair.

The Prince of Wales will visit the Sussex County Hospital on Saturday, the 29th inst., and has consented to receive purses of not less than £5 in aid of the building fund of the institution.

The annual general meeting of the Governors of the Royal Hospital, Richmond, will be held in the Princess May's Ward of the hospital on Saturday, February 29th, at four p.m.

Mr. William Turner, M.B., F.R.C.S., L.R.C.P., has been appointed one of the visiting surgeons to the Seamen's Hospital at the Royal Victoria and Albert Docks, which is a branch of the Seamen's Hospital Society (Dreadnought) at Greenwich.

Through the will of the late Lady Gregory, better known as Mrs. Stirling, several London hospitals are to receive handsome legacies. Amongst these are the Westminster, the Middlesex, the Charing Cross, St. Mary's, the Hospital for Sick Children, the Hospital for Women, the Samaritan Free Hospital for Women and Children, and the Victoria Hospital for Children, each of which will receive £250.

The managers of the Metropolitan Sunday Fund should feel encouraged by the Pecksniffian patronage which is bestowed upon them in "Fry's Guide to London Charities." Writing of the contributions of 1895, the Editor says, "God grant that the Hospital Sunday Fund may maintain this important increased subscription, *to whatever cause it is due*, and that it may not prove *merely* an exceptional one." The italics are our own.

The Hospital for Consumption and Diseases of the Chest at Belfast is about to be known as the Foster Green Hospital for Consumption. Mr. Foster Green, who has already shown the greatest interest in the old institution, has now presented funds which will enable it to be transferred to a new site. He has purchased the Fortbreda property, which has cost a sum of £11,000, and in order to obviate difficulties has obtained the consent of the authorities of the original institution to alter the whole constitution in merging it in the new establishment. The Belfast Hospital for Consumption has prided itself upon economy in expenditure, the average cost per bed having reached only £31 6s. 6d. For a consumption hospital this expenditure is, of course, remarkably small, but the committee of management are satisfied that the comfort of the patients has not been sacrificed. It is not expected that the rehabilitated hospital can be carried on at an equally small cost, and it is hoped that the inhabitants of Belfast will increase and not diminish their annual subscriptions and donations to the hospital. A system obtains in connection with the hospital which, as we believe it to be unique, we mention. This is, that subscribers are invited to send at the beginning of each month one pound of some useful article of domestic consumption, such as sugar, tea, &c. The invitation has met with a hearty response, and the supplies of the hospital are thereby largely replenished.

